# The mitochondrial genome of a social wasp, *Vespa simillima simillima* (Hymenoptera: Vespidae)

**DOI:** 10.1080/23802359.2019.1699458

**Published:** 2019-12-13

**Authors:** Moon-Bo Choi, Young-Ho Ha, Il-Kown Kim, Seung Hwan Oh, Chang-Jun Kim

**Affiliations:** a School of Applied Biosciences, Kyungpook National University, Daegu, Republic of Korea;; b Division of Forest Biodiversity, Korea National Arboretum, Pocheon, Republic of Korea;; c Division of DMZ Native Botanic Garden, Korea National Arboretum, Pocheon, Republic of Korea

**Keywords:** Mitochondrial genome, *Vespa simillima simillima*, phylogeny

## Abstract

We analyzed the complete mitochondrial genome of a social wasp, *Vespa simillima simillima* from South Korea prior to a systematic study on Korean Vespidae. The mitogenome is 16,740 bp in length, includes 13 protein-coding genes (PCGs), 22 tRNAs, 2 rRNAs, and a 228 bp short A + T-rich region. The overall base composition is 82.0% AT and 18.0% GC. The maximum-likelihood analysis suggested that *V*. *s*. *simillima* is closely related to *V*. *bicolor*, another species of Vespidae.

*Vespa simillima* is commonly distributed in the Northeast Asia, and the subspecies *simillima* is especially prevalent in Japan, Far East Russia and Korea (Matsuura and Yamane 1984; Archer 2012). Two subspecies of *V*. *simillima* are known from Korea, but the subspecies *simillima* is very common throughout the mainland, and *xanthoptera* is distributed only in southern Jeju Island (Choi et al. 2013). *Vespa simillima simillima* is one of the *Vespa* species frequently found in urban areas nowadays, and is capable of building the largest nest, housing 1000–2000 adults, among Korean *Vespa* species (Choi and Moon 2005). In recent years, there has been an increase in social wasp sting accidents in Korea, and this species is also frequently found in urban areas (Choi and Moon 2005). In particular, the rate of wasp nest removal is currently about 4–10% of all wasps, and the harmful effect by the wasp gradually increases (Choi et al. 2019). Therefore, it is essential to understand the genetic and ecological diversity of *Vespa* species, which can be crucial key factors for rapid response to prevent accidents (Matsuura and Yamane, 1984).

A voucher specimen (2018_CJ4) collected from Odaesan National Park, Gangwon Province, South Korea (37°42′11.2″N, 128°36′07.4″E) in September 2018, is deposited in the Korea National Arboretum, Pocheon, South Korea (KNA).

In this study, the complete mitochondrial genome of the Korean species, *V*. *simillima simillima* was sequenced using Illumina MiSeq. A total of 16,272,836 raw reads were obtained and trimmed (error probability limit: 0.01) using the Geneious v. 10.2.3 (Kearse et al. 2012). The secondary structure prediction of tRNAs except for tRNAAsn and tRNASer annotated with the improved Mitochondrial Genome annotation (MITOS) webserver (Bernt et al. 2013: http://mitos.bioinf.uni-leipzig.de/), and the secondary structures of tRNA genes were analyzed by compariing with the nucleotide sequences of other insect tRNA sequences.

Maximum likelihood analysis of Vespoidea was conducted based on concatenated 13 mitochondrial coding genes using IQTREE v.1.6.8 (Nguyen et al. 2015) with 1000 bootstrap (BS) replications. *Formica fusca* in Formicidae was used as an outgroup. The analysis constructed a robust phylogenetic tree with high supports for all nodes (Figure 1). The tree indicated that the genus *Vespa* is monophyletic, and *V*. *s*. *simillima* is a sister taxon to *V*. *bicolor* among the species used in the analysis. The mitogenome data of the species will be used for genetic diversity and phylogenetic studies of Vespoidea.

**Figure 1.**
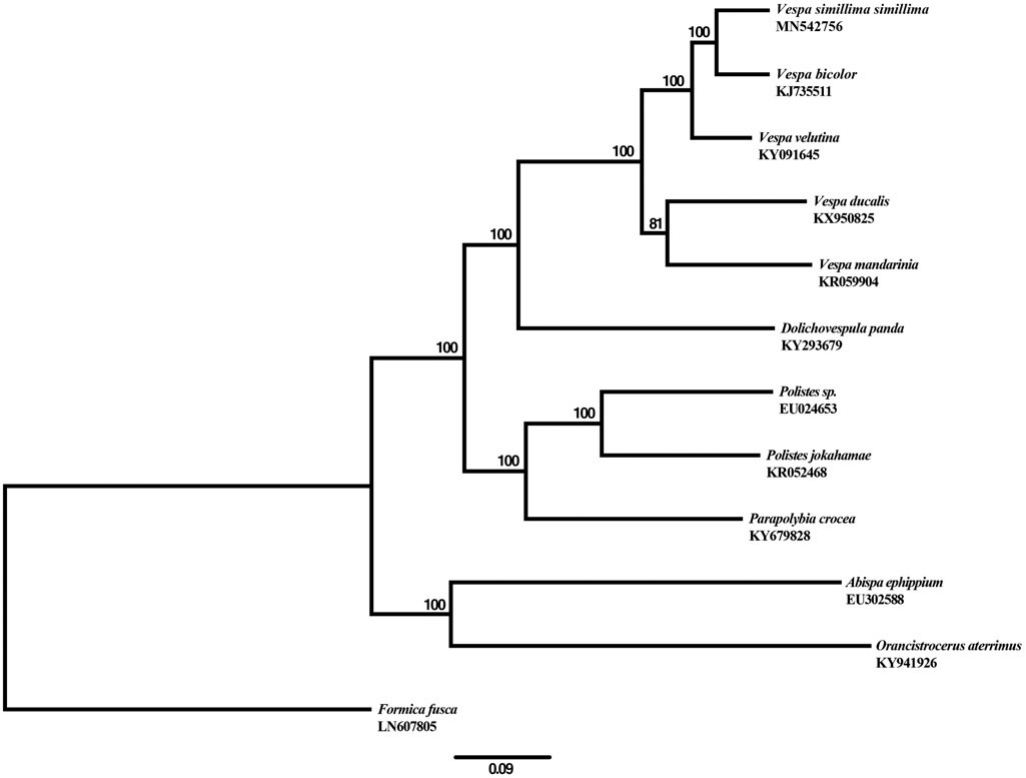
A maximum-likelihood phylogenetic tree inferred from the sequences of 13 PCGs in the mitochondrial genomes of 11 vespoid species including *V*. *s*. *simillima*. Numbers in the nodes are the bootstrap values from 1000 replicates. *Formica fusca* was used as an outgroup. Alphanumeric terms indicate the GenBank accession numbers.

## Nucleotide sequence accession number

The complete mitochondrial genome sequence of *Vespa simillima simillima* has been assigned GenBank accession number MN542756.
